# Expression of a bacterial 3‐dehydroshikimate dehydratase reduces lignin content and improves biomass saccharification efficiency

**DOI:** 10.1111/pbi.12310

**Published:** 2015-01-13

**Authors:** Aymerick Eudes, Noppadon Sathitsuksanoh, Edward E. K. Baidoo, Anthe George, Yan Liang, Fan Yang, Seema Singh, Jay D. Keasling, Blake A. Simmons, Dominique Loqué

**Affiliations:** ^1^ Joint BioEnergy Institute Emeryville CA USA; ^2^ Physical Biosciences Division Lawrence Berkeley National Laboratory Berkeley CA USA; ^3^ Sandia National Laboratory Livermore CA USA; ^4^ Department of Bioengineering Department of Chemical & Biomolecular Engineering University of California Berkeley CA USA

**Keywords:** cell wall, lignin, QsuB, saccharification, lignin polymerization degree, bioenergy

## Abstract

Lignin confers recalcitrance to plant biomass used as feedstocks in agro‐processing industries or as source of renewable sugars for the production of bioproducts. The metabolic steps for the synthesis of lignin building blocks belong to the shikimate and phenylpropanoid pathways. Genetic engineering efforts to reduce lignin content typically employ gene knockout or gene silencing techniques to constitutively repress one of these metabolic pathways. Recently, new strategies have emerged offering better spatiotemporal control of lignin deposition, including the expression of enzymes that interfere with the normal process for cell wall lignification. In this study, we report that expression of a 3‐dehydroshikimate dehydratase (QsuB from *Corynebacterium glutamicum*) reduces lignin deposition in *Arabidopsis* cell walls. QsuB was targeted to the plastids to convert 3‐dehydroshikimate – an intermediate of the shikimate pathway – into protocatechuate. Compared to wild‐type plants, lines expressing QsuB contain higher amounts of protocatechuate, *p*‐coumarate, *p*‐coumaraldehyde and *p*‐coumaryl alcohol, and lower amounts of coniferaldehyde, coniferyl alcohol, sinapaldehyde and sinapyl alcohol. 2D‐NMR spectroscopy and pyrolysis‐gas chromatography/mass spectrometry (pyro‐GC*/*MS) reveal an increase of *p*‐hydroxyphenyl units and a reduction of guaiacyl units in the lignin of QsuB lines. Size‐exclusion chromatography indicates a lower degree of lignin polymerization in the transgenic lines. Therefore, our data show that the expression of QsuB primarily affects the lignin biosynthetic pathway. Finally, biomass from these lines exhibits more than a twofold improvement in saccharification efficiency. We conclude that the expression of QsuB in plants, in combination with specific promoters, is a promising gain‐of‐function strategy for spatiotemporal reduction of lignin in plant biomass.

## Introduction

Plant cells walls are the primary source of terrestrial biomass and mainly consist of cellulosic and hemicellulosic polysaccharides impregnated with lignins. Lignins are polymers of *p*‐hydroxycinnamyl alcohols (i.e. monolignols), which are synthesized inside the cells, exported to the cell wall and ultimately undergo oxidative polymerization via laccase and peroxidase activities. The main monolignols – *p*‐coumaryl, coniferyl and sinapyl alcohols – give rise to the *p*‐hydroxyphenyl (H), guaiacyl (G) and syringyl (S) lignin units, respectively (Boerjan *et al*., 2003). Lignification generally confers mechanical strength and hydrophobicity in tissues that develop secondary cell walls, such as sclerenchyma (i.e. fibres) and xylem vessels. In addition to its essential role for upright growth, lignin also serves as a physical barrier against pathogens that degrade cell walls (Boudet, 2007).

Lignocellulosic biomass is used for pulp and paper manufacture, ruminant livestock feeding, and more recently has been considered an important source of simple sugars for fermentative production of intermediate or specialty chemicals and biofuels (Keasling, 2010). It is well‐documented that lignin in plant biomass negatively affects pulp yield, forage digestibility and polysaccharide saccharification (Baucher *et al*., 2003; Chen and Dixon, 2007; Taboada *et al*., 2010). This has prompted major interest in developing a better understanding of lignin biosynthesis to reduce biomass recalcitrance by modifying lignin content and/or composition.

**Figure 1:**
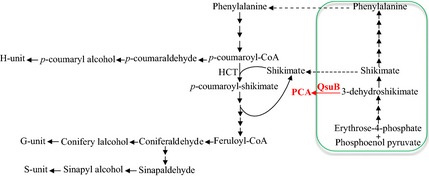
The lignin biosynthetic pathway and heterologous expression of bacterial 3‐dehydroshikimate dehydratase. HCT, hydroxycinnamoyl‐coenzyme A shikimate/quinate hydroxycinnamoyltransferase; QsuB, 3‐dehydroshikimate dehydratase from *Corynebacterium glutamicum*; PCA, protocatechuate.

The shikimate pathway, which is located in plastids in plants, provides a carbon skeleton for the synthesis of phenylalanine, the precursor of the cytosolic phenylpropanoid pathway responsible for the biosynthesis of monolignols (Figure 1). All the metabolic steps and corresponding enzymes for both pathways are known and well‐conserved across land plants (Fraser and Chapple, 2011; Tohge *et al*., 2013; Umezawa, 2010). Classic approaches to lignin reduction have relied on genetic modifications, such as transcript reduction and allelic variation of specific genes from the phenylpropanoid pathway (Li *et al*., 2008; Vanholme *et al*., 2008). However, these strategies often result in undesired phenotypes – including dwarfism, sterility and increased susceptibly to environmental stresses – due to loss of cell wall integrity, depletion of other phenylpropanoid‐related metabolites, accumulation of pathway intermediates or the constitutive activation of defence responses (Bonawitz and Chapple, 2013; Voelker *et al*., 2011). Such negative effects are unfortunately difficult to avoid because of the nontissue specificity of the strategies employed: allelic variations are transmitted to every cell of the plant during cell divisions, and small interfering RNAs generated for gene silencing generally move from cell‐to‐cell and over long distance in vegetative tissues (Brosnan and Voinnet, 2011).

Alternatively, there are novel and promising gain‐of‐function strategies that involve expression of specific proteins to reduce the production of the three main monolignols or change their ratios. Using specific promoters with restricted expression patterns, these strategies would enable the alteration of lignin at later developmental stages or, for example, only in certain tissues such as fibres – without compromising the functionality of conductive vessels for the transport of water (Voelker *et al*., 2011). Examples of such expressed proteins are transcription factors that act as negative regulators of lignin biosynthesis (Fornalé *et al*., 2010; Iwase *et al*., 2009; Shen *et al*., 2012; Yan *et al*., 2013); enzymes that produce alternative lignin monomers (Eudes *et al*., 2012; Wilkerson *et al*., 2014); engineered enzymes that modify monolignols into their nonoxidizable forms (Zhang *et al*., 2012); or proteins that mediate the post‐transcriptional degradation of enzymes from the lignin biosynthetic pathway (Zhang *et al*., 2014).

In this study, we report for the first time on the expression of a bacterial 3‐dehydroshikimate dehydratase in *Arabidopsis* (Teramoto *et al*., 2009). We selected QsuB from *C*. *glutamicum* and targeted it to the plastids to convert the shikimate precursor 3‐dehydroshikimate into protocatechuate (Figure 1), with the aim of reducing lignin content and modifying its composition as shikimate is required for lignin biosynthesis. Metabolomic analysis of plants expressing QsuB revealed higher amounts of *p*‐coumarate and of the two direct precursors of H‐lignin units: *p*‐coumaraldehyde and *p*‐coumaryl alcohol. Conversely, the direct precursors of G and S units – coniferaldehyde, coniferyl alcohol, sinapaldehyde and sinapyl alcohol – were reduced. Lignin content was severely reduced in these transgenic lines and exhibited an enrichment of H units at the expense of G units and a lower polymerization degree. Compared to those of wild‐type plants, cell walls from lines expressing QsuB released significantly higher amounts of simple sugars after cellulase treatment and required less enzyme for saccharification. Collectively, these results support the hypothesis that expression of a plastidic QsuB affects the lignin biosynthetic pathway.

## Results

### Targeted expression of QsuB in *Arabidopsis*

**Figure 2:**
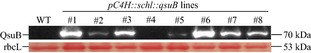
QsuB expression in *Arabidopsis* stems. Detection by Western blot of QsuB tagged with the AttB2 peptide (approximate size 70 kDa) using the ‘universal antibody’ and stem proteins from eight independent 6‐week‐old homozygous *pC4H::schl::qsuB* T3 transformants. A stem protein extract from wild type was used as a negative control (WT), and a Ponceau staining of Rubisco large subunit (rbcL) is shown as a loading control.

A sequence encoding QsuB was cloned downstream of the sequence encoding for a plastid‐targeting signal peptide (SCHL) for expression in plastids. Using transient expression in tobacco, we first confirmed that QsuB was correctly targeted to the plastids by analysing its subcellular localization when fused at the C‐terminus to a YFP marker (Figure S1). The *schl*‐*qsuB* sequence was cloned downstream of the *Arabidopsis C4H* promoter for expression in lignifying tissues of *Arabidopsis*. Western blot analysis confirmed that QsuB was expressed in stems of several T3 plants homozygous for the *pC4H::schl::qsuB* (thereafter *C4H::qsuB*) construct (Figure 2). Based on the migration of molecular weight markers, QsuB was detected at around 70 kDa, which corresponds to the theoretical size of its native sequence after cleavage of the chloroplast transit peptide (Figure 2). Four homozygous lines with different QsuB expression levels (*C4H::qsuB‐1*, ‐*3*, ‐*6* and ‐*7*) were selected for biomass measurement. Although a height reduction was observed for these lines, only *C4H::qsuB‐1* showed a slight decrease (−18%) of biomass yield (Table 1).

**Table 1:** Height and dry weight of the main inflorescence stem of senesced mature wild‐type (WT) and *pC4H::schl::qsuB* (*C4H::qsuB*) plants. Number, *n,* of plants analysed

Plant line	Height (cm) Mean ± SE	Dry weight (mg) Mean ± SE	*n*
WT	47.3 ± 0.8	271.0 ± 11.1	24
*C4H::qsuB‐1*	36.6 ± 1.0**	221.3 ± 11.0*	20
*C4H::qsuB‐3*	38.8 ± 0.7**	244.4 ± 13.4	20
*C4H::qsuB‐6*	35.9 ± 0.9**	254.1 ± 12.7	20
*C4H::qsuB‐7*	41.0 ± 0.9**	251.3 ± 17.4	20

Asterisks indicate significant differences from the wild type using the unpaired Student's t‐test (*P <* *0.005; **P <* *0.001).

### Metabolite analysis of *C4H::qsuB* lines

Methanol‐soluble metabolites from stems of the four homozygous *C4H::qsuB* lines were extracted for analysis (Table 2, Figure S2). Compared to wild‐type plants, protocatechuate content was increased 67‐ to 113‐fold in the transgenic lines. However, no significant reduction was observed for the content of several metabolites derived from the shikimate pathway such as salicylate and aromatic amino acids (i.e. phenylalanine, tyrosine and tryptophan). Interestingly, several metabolites from the phenylpropanoid pathway were increased in the transgenic lines; *p*‐coumaraldehyde and *p*‐coumaryl alcohol, the two direct precursors of H‐lignin units, were increased 5.7–16.4‐fold and 12.2–13.7‐fold, respectively. Similarly, *p*‐coumarate content was increased 6.4–9.5‐fold compared to wild type. In contrast, the direct precursors of G‐ and S‐lignin units were negatively altered in transgenic lines. Coniferaldehyde and coniferyl alcohol were reduced by 33–50% and 36–68%, respectively. Sinapaldehyde and sinapyl alcohol were decreased by 45–77% and 73–87%, with the exception of line *C4H::qsuB‐1* which showed no significant difference for sinapaldehyde compared to wild type (Table 2).

**Table 2:** Quantitative analysis of methanol‐soluble metabolites in stems from 5‐week‐old wild‐type (WT) and *pC4H::schl::qsuB* (*C4H::qsuB*) plants. Values in brackets are the SE from four biological replicates (*n *=* *4)

Metabolites	Mean (^α^μg/g or ^β^ng/g fresh weight)				
	WT	*C4H::qsuB‐1*	*C4H::qsuB‐3*	*C4H::qsuB‐6*	*C4H::qsuB‐7*
Protocatechuate^α^	1.2 (0.6)	110.4 (15.4)***	133.4 (14.0)***	79.7 (15.9)***	118.7 (16.2)***
Tryptophan^α^	3.5 (0.6)	2.9 (0.1)	3.4 (0.5)	3.1 (0.7)	3.0 (0.3)
Phenylalanine^α^	4.9 (0.5)	4.9 (0.9)	4.1 (0.5)	4.1 (0.4)	4.5 (0.3)
Tyrosine^α^	7.3 (1.0)	6.7 (0.6)	8.2 (0.5)	6.7 (1.3)	6.4 (0.6)
Salicylate^β^	755.4 (33.1)	762.9 (59.8)	732.7 (54.4)	695.6 (25.5)	685.9 (26.9)
*p‐*coumaraldehyde^β^	0.8 (0.2)	4.8 (1.6)*	11.7 (2.2)**	8.7 (0.7)**	13.9 (3.3)**
*p*‐coumaryl alcohol^β^	13.2 (1.4)	181.1 (20.9)***	180.3 (52.4)*	160.4 (46.1)*	175.9 (33.0)**
*p*‐coumarate^β^	5.9 (0.4)	55.9 (8.7)**	47.8 (13.4)*	41.7 (13.5)*	37.6 (6.5)**
Coniferaldehyde^β^	18.0 (1.4)	12.0 (1.5)*	9.6 (2.4)*	9.1 (1.1)**	11.3 (1.5)*
Coniferyl alcohol^β^	792.6 (87.0)	504.5 (70.1)*	363.3 (101.9)*	255.0 (26.3)**	325.4 (7.3)**
Sinapaldehyde^β^	14.7 (1.6)	12.8 (1.5)	8.1 (2.7)*	3.4 (1.3)**	5.7 (1.2)**
Sinapyl alcohol^β^	2752.8 (334.9)	731.5 (101.1)**	357.4 (123.8)***	350.6 (171.7)***	540.1 (57.8)***

Asterisks indicate significant differences from the wild type using the unpaired Student's t‐test (*P <* *0.05; **P <* *0.005; ***P <* *0.001).

Cell wall‐bound *p*‐coumarate and ferulate released from cell wall residues by mild alkaline hydrolysis were also analysed (Table 3). The content of *p*‐coumarate was significantly increased in the *C4H::qsuB* lines (1.75–3‐fold)*,* whereas ferulate was reduced (1.8–2.9‐fold). In addition, bound *p*‐coumaraldehyde could be detected in cell wall samples from the transgenic lines but not in those from wild type (Table 3).

**Table 3:** Quantitative analysis of cell wall‐bound aromatics in stems from extractive‐free senesced mature wild‐type (WT) and *pC4H::schl::qsuB* (*C4H::qsuB*) plants. Values are means of three biological replicates (*n *=* *3)

Plant line	Mean ± SE (^α^μg/g or ^β^ng/g cell wall)		
	*p*‐coumarate^α^	Ferulate^α^	*p*‐coumaraldehyde^β^
WT	5.4 ± 0.6	41.8 ± 4.3	ND
*C4H::qsuB‐1*	9.4 ± 1.2*	14.5 ± 0.8**	47.6 ± 13.0**
*C4H::qsuB‐3*	15.4 ± 1.9**	19.3 ± 1.3**	64.8 ± 6.6**
*C4H::qsuB‐6*	16.5 ± 2.6*	20.8 ± 2.4*	96.5 ± 19.0**
*C4H::qsuB‐7*	14.5 ± 0.9**	22.9 ± 1.8*	62.1 ± 0.4**

ND, not detected.

Asterisks indicate significant differences from the wild type using the unpaired Student's t‐test (*P <* *0.05; **P <* *0.01).

### Lignin content and monomeric composition in *C4H::qsuB* lines

The Klason method was used to measure the lignin content: a reduction ranging from 45% (*C4H::qsuB‐7*) to 52% (*C4H::qsuB‐1*) was observed in stems of the *C4H::qsuB* lines compared to wild type (Table 4). Cell wall material from stems of wild‐type and *C4H::qsuB* lines was analysed by pyro‐GC*/*MS for the determination of the lignin monomer composition. For each line, identification and relative quantification of the pyrolysis products derived from H, G or S units allowed determination of H/G/S ratios (Table 4, Table S1). Compared to wild type, the relative amount of H units is increased between 3.3‐fold (*C4H::qsuB‐3*) and 6‐fold (*C4H::qsuB‐6*) in transgenics. The relative amount of S units is moderately increased 1.3–1.5‐fold, whereas that of G units is reduced 1.5–1.8‐fold in the *C4H::qsuB* lines.

**Table 4:** Lignin content and composition in senesced mature stems from wild‐type (WT) and *pC4H::schl::qsuB* (*C4H::qsuB*) plants. Values in brackets are the SE from three biological replicates (*n *=* *3)

	Klason lignin (mg/g cell wall)	%H	%G	%S
WT	177.8 (18.2)	3.3 (0.2)	64.1 (1.9)	32.6 (2.0)
*C4H::qsuB‐1*	85.0 (4.6)**	15.5 (0.2)**	38.9 (0.6)**	45.6 (0.5)*
*C4H::qsuB‐3*	95.4 (1.5)**	10.8 (0.4)**	39.4 (1.2)**	49.8 (0.9)*
*C4H::qsuB‐6*	91.4 (6.4)**	20.0 (1.0)**	36.9 (2.8)*	43.1 (3.5)*
*C4H::qsuB‐7*	97.8 (1.2)**	12.8 (1.8)*	43.8 (1.3)**	43.4 (1.9)*

Asterisks indicate significant differences from the wild type using the unpaired Student's t‐test (*P <* *0.05, **P <* *0.01).

**Figure 3:**
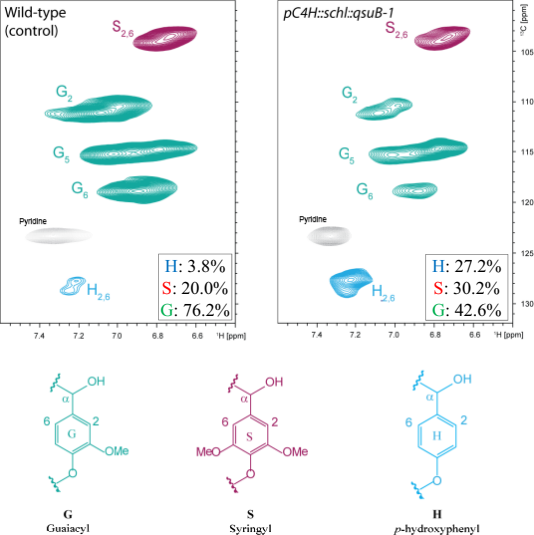
Partial short‐range ^13^C–^1^H (HSQC) spectra (aromatic region) of cell wall material from mature senesced stems of wild‐type (WT) and *pC4H::schl::qsuB‐1* plants. Lignin monomer ratios are provided on the figures.

NMR (2D ^13^C–^1^H‐correlated, HSQC) spectra of cell wall material from wild‐type and *C4H::qsuB‐1* plants were also obtained for determination of lignin composition and structure. Analysis of the aromatic region of the spectra confirmed the higher relative amount of H units in *C4H::qsuB*‐1 (27.2%) compared to wild type (3.8%), as well as a reduction of G units (Figure 3). Moreover, analysis of the aliphatic region of the spectra indicated a diminution of phenylcoumaran (β‐5) and resinol (β‐β) linkages in the lignin of the *C4H::qsuB‐1* line (Figure S3).

### Lignins from *C4H::qsuB* plants have a lower polymerization degree

**Figure 4:**
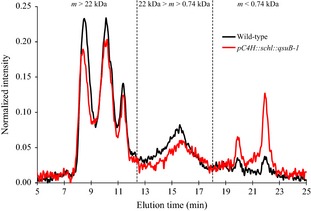
Polydispersity of cellulolytic enzyme lignins from wild‐type and *pC4H::schl::qsuB*‐*1* plants. Cellulolytic enzyme lignins were purified from mature senesced stems of wild‐type (black line) and *pC4H::schl::qsuB‐1* (red line) plants and analysed for polydispersity by size‐exclusion chromatography (SEC). SEC chromatograms were obtained using UV‐F fluorescence (Ex_250_/Em_450_). m, molecular mass.

Lignin fractions were isolated from wild‐type and *C4H::qsuB‐1* plants for analysis of their polydispersity using size‐exclusion chromatography (SEC). Elution profiles acquired by monitoring UV‐F fluorescence of the dissolved lignin revealed differences between wild‐type and the transgenic line (Figure 4). The total area of the three mass peaks, corresponding to the largest lignin fragments detected between 7.8 and 12.5 min, was significantly reduced in *C4H::qsuB‐1* compared to wild type. Similarly, intermediate molecular mass material, which elutes in a fourth peak between 12.5 and 18 min, was also less abundant in the *C4H::qsuB* line. Conversely, the area corresponding to the smallest lignin fragments, detected between 18 and 23.5 min, was increased in the transgenic line. These results demonstrate a reduction in the degree of polymerization of lignins purified from plants expressing QsuB compared to that of wild type.

### Biomass from *C4H::qsuB* lines shows improved saccharification

**Figure 5:**
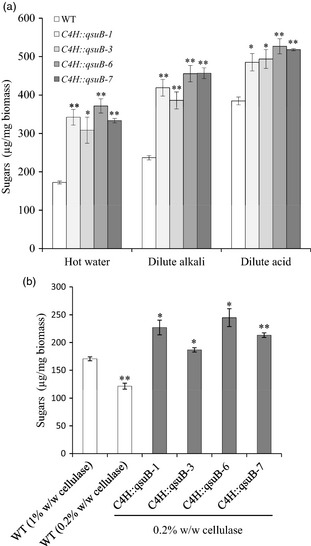
Saccharification of biomass from mature senesced stems of wild‐type (WT) and *pC4H::schl::qsuB* (*C4H::qsuB*) lines. (a) Amounts of sugars released from biomass after various pretreatments and 72‐h enzymatic digestion with cellulase (1% w/w). Values are means ± SE of four biological replicates (*n *=* *4). Asterisks indicate significant differences from the wild type using the unpaired Student's t‐test (*P <* *0.05; **P <* *0.005). (b) Amounts of sugars released from biomass after hot water pretreatment and 72‐h enzymatic digestion using two different cellulase loadings (1% or 0.2% w/w). Values are means ± SE of four biological replicates (*n *=* *4). Asterisks indicate significant differences from the wild type at 1% cellulase loading using the unpaired Student's t‐test (*P <* *0.05; **P <* *0.005).

Saccharification assays on stem material were conducted to evaluate the cell wall recalcitrance of the *C4H::qsuB* lines. As shown in Figure 5a, higher amounts of sugars were released after 72 h enzymatic hydrolysis of biomass from the *C4H::qsuB* lines compared to those of wild type in all pretreatments tested. Saccharification improvements ranged between 79–116% after hot water, 63–93% after dilute alkali and 26–37% after dilute acid pretreatments (Figure 5a). Moreover, similar saccharification experiments using hot water‐pretreated biomass, at 5× lower cellulase loadings, revealed that biomass from all *C4H::qsuB* lines releases more sugar than that of wild type hydrolysed with a typical enzyme loading (Figure 5b). Taken together, these data demonstrate that cellulose from the *C4H::qsuB* lines is less recalcitrant to cellulase digestion and requires a lower amount of enzyme to be converted into high yields of fermentable sugars.

## Discussion

Gain‐of‐function strategies have several advantages for the manipulation of metabolic pathways. For example, they can be used to bioengineer lignin deposition in plants via better spatiotemporal control of monolignol production in lignifying cells and to adjust lignin composition and its biophysical properties (Eudes *et al*., 2014). Therefore, identification of proteins in which in planta‐expression results in modifications of lignin content or composition is of particular interest and presents novel opportunities. In this work, we demonstrate that expression of the 3‐dehydroshikimate dehydratase QsuB in plastids leads to drastic reduction and compositional changes of lignin in *Arabidopsis* (Table 4). As a result, biomass from these transgenic plants exhibits much higher saccharification efficiency after pretreatment (Figure 5a), which is a highly desired trait for several agro‐industries and the bioenergy sector. Moreover, the efficiency of this approach to decrease lignin content in plant biomass allows a reduction of hydrolytic enzyme loadings by at least fivefold, while retaining greater saccharification potential than control plants hydrolysed at standard enzyme loading (Figure 5b). Consequently, the transfer of this technology to energy crops should have a great impact on the cost‐effectiveness of cellulosic biofuels production, because enzyme cost is the major barrier in this process (Klein‐Marcuschamer *et al*., 2012).

In this study, as a proof of concept, we used the promoter of the *AtC4H* gene to ensure strong QsuB expression in all lignifying tissues of the plant. This resulted in a slight decrease of plant height for all the lines, but no significant reductions in biomass yield except for that of one transgenic line, which expressed QsuB strongly (Table 1; Figure 2) and exhibited – in some stem transverse sections (Figure S4) – evidence of vessel collapse that could impair xylem conductivity (Voelker *et al*., 2011). Nevertheless, our strategy offers the potential to overcome these defects by selecting more stringent promoters (e.g. fibre‐specific) that would exclude QsuB expression from xylem‐conductive elements (Eudes *et al*., 2014; Yang *et al*., 2013). Other particular phenotypes such as increased branching or thicker stems, which would explain the unaffected biomass yield despite a reduced height in some transgenic lines, were not observed (Figure S5). Hypothetically, the biomass from the transgenic lines could be denser than that of wild type as previously shown in plants with lower lignin content (Nuopponen *et al*., 2006). Moreover, translation of our technology from model plant to crops is expected to be straightforward: it is based solely on the expression of QsuB and does not require any particular genetic backgrounds, and the lignin and shikimate pathways are well‐conserved among vascular plants.

A direct consequence of QsuB expression is the accumulation of protocatechuate in the biomass of transgenic plants. Considering the beneficial properties of protocatechuate in the bio‐based chemical industry, such de novo production adds extra commercial value to the biomass of plants expressing QsuB (Linger *et al*., 2014; Otsuka *et al*., 2006). Much higher amounts of protocatechuate were recovered after acid treatment of the methanol‐soluble extracts from transgenic plants (data not shown), which suggests its conjugation in the cytosol after export from the plastids. Interestingly, in stems of 5‐week‐old plants, QsuB expression did not affect the overall level of metabolites derived from the shikimate pathway such as aromatic amino acids and salicylate, suggesting that plastidic 3‐dehydroshikimate is not limiting (Table 2). Although an alternative cytosolic pathway for salicylate has been described (Chen *et al*., 2009), the *de novo* biosynthesis of aromatic amino acids outside plastids remains undefined (Maeda and Dudareva, 2012), suggesting that 3‐dehydroshikimate is not limiting at least for the biosynthesis of aromatic amino acids in plants expressing QsuB. On the other hand, a build‐up of the pool of *p*‐coumarate, *p*‐coumaraldehyde and *p*‐coumaryl alcohol, the precursors of H‐lignin units, was observed in the transgenic lines (Table 2 and Figure S2).

Analysis of the lignin monomeric composition – using 2D NMR spectroscopy and pyro‐GC*/*MS – unequivocally demonstrated an increase in H units in plants expressing QsuB (Figure 3; Tables 4 and S1). These data could explain the reduced degree of polymerization of these lignins, which has been previously observed in various lignin mutants that exhibit high content of H units, incorporation of which typically slows or stops lignin‐chain elongation (Sangha *et al*., 2014; Ziebell *et al*., 2010; Figure 4). Therefore, reduced lignin–polysaccharide cross‐linking within the biomass of the transgenic lines is expected, and this could contribute to its superior enzymatic digestibility (Ralph *et al*., 2004).

A low lignin content rich in H units and higher S/G corresponds to a phenotype previously characterized in plants down‐regulated for hydroxycinnamoyl‐CoA shikimate/quinate hydroxycinnamoyl transferase (HCT), *p*‐coumarate 3‐hydroxylase (C3H) or caffeoyl shikimate esterase (CSE) (Ralph *et al*., 2006; Vanholme *et al*., 2013; Ziebell *et al*., 2010). Moreover, reduction of HCT activity results in the accumulation of free and bound *p*‐coumaraldehyde in cucumber and of *p*‐coumarate in alfalfa, presumably due to the build‐up of coumaroyl‐CoA (Gallego‐Giraldo *et al*., 2011; Varbanova *et al*., 2011). This suggests that an alteration of these biosynthetic steps has occurred in the *C4H::qsuB* lines. However, no particular reduction of transcript abundance for *HCT*,* C3H* and *CSE* was observed in the transgenic lines compared to wild type (Figure S6). A possible explanation is that QsuB activity, which consumes 3‐dehydroshikimate in lignifying tissues, affects indirectly the amount of shikimate available for HCT in the cytosol. Although some enzymes of the shikimate pathway exist in the cytosol, there is so far no evidence for a complete alternative extra‐plastidial shikimate biosynthetic pathway. Instead, a yet‐unidentified transporter probably mediates the export of shikimate from the plastid to the cytosol (Maeda and Dudareva, 2012). If such transport system is only active at a narrow range of concentrations, a reduction of shikimate content in plastids (as anticipated in plants expressing QsuB) would compromise its export to the cytosol. Moreover, it is possible that the large amount of protocatechuate generated by QsuB activity in plastid competes with shikimate export. The distribution of shikimate between plastids and the cytosol is still poorly understood, and shikimate levels were below the detection limit in our stem extracts from wild type and transgenic plants. Alternatively, because previous studies reported a substrate flexibility of HCTs (Moglia *et al*., 2010; Sander and Petersen, 2011), the large accumulation of protocatechuate could act as competitive inhibitor of HCT, thus limiting the synthesis of coumaroyl shikimate required for the production of G‐ and S‐lignin units.

## Experimental procedures

### Plant material and growth conditions

*Arabidopsis thaliana* (ecotype Columbia, Col‐0) seeds were germinated directly on soil. Growing conditions were 150 μmol/m^2^/s, 22 °C, 60% humidity and 10 h of light per day. Selection of T2 and identification of T3 homozygous transgenic plants were made on Murashige and Skoog vitamin medium (PhytoTechnology Laboratories, Shawnee Mission, KS, USA), supplemented with 1% sucrose, 1.5% agar and 50 μg/mL kanamycin.

### Generation of binary vectors

The promoter *p35S*, with a single enhancer, was amplified by PCR from pRT100 with phosphorylated primers F‐p35S (5′‐GTCAACATGGTGGAGCACGACAC‐3′) and R‐p35S (5′‐CGAGAATCTAGATTGTCCTCTCCAAATGAAATGAACTTC‐3′), and cloned into a *Sma*I‐digested dephosphorylated pTkan vector (Yuan *et al*., 2009) to generate a pTKan‐*p35S* vector. Subsequently, a GW‐YFP cassette was extracted from the pX‐YFP vector (Kim *et al*., 2009) by *Xho*I/*Spe*I digestion and ligated into a *Xho*I/*Spe*I‐digested pTKan‐*p35S* vector to generate the pTkan‐*p35S*‐GWR1R2‐YFP vector.

A chimeric DNA construct was synthesized (GenScript, Piscatway, NJ, USA): it was flanked by the gateway sequences attB4r (5′‐end) and attB3r (3′‐end), and contained, in the following order, the *tG7* terminator; the restriction sites *Sma*I, *Kpn*I, *Hin*dIII and *Xho*I; a 2.9‐Kb sequence corresponding to the *Arabidopsis* C4H promoter (*pC4H*); and a sequence encoding a plastid‐targeting signal (SCHL; Lebrun *et al*., 1992). This attB4r‐*tG7*‐*pC4H*‐*schl*‐attB3r construct was then subcloned into the Gateway pDONR221‐P4rP3r entry vector by BP recombination (Life technologies, Foster City, CA, USA) to generate pENTR‐L4‐tG7‐*pC4H*‐*schl*‐L3. An LR recombination reaction was performed with pTkan‐*pIRX5*‐GW (Eudes *et al*., 2012), pENTR‐L1‐*pLac*‐lacZalpha‐L4 (Life technologies), pENTR‐L3‐*pLac*‐Tet‐L2 (Life technologies) and pENTR‐L4‐*tG7*‐*pC4H*::*schl*‐L3. The obtained construct was subsequently digested by *Sma*I to remove the *pLac*‐lacZalpha and *tG7* fragments. The *pLac*‐Tet fragment was replaced by the gateway cassette using BP recombination to generate the pTKan‐*pC4H*::*schl*‐GWR3R2 vector.

### Generation of a pTkan‐*pC4H*::*schl*::*qsuB* plasmid and plant transformation

A gene sequence encoding QsuB from *C. glutamicum* (GenBank Accession Number YP_001137362.1) without stop codon and flanked with the Gateway *att*B3 (5′‐end) and *att*B2 (3′‐end) recombination sites was synthesized for expression in *Arabidopsis* (GenScript) and cloned into the Gateway pDONR221‐P3P2 entry vector by BP recombination (Life technologies). A sequence‐verified entry clone was LR recombined with the pTKan‐*pC4H::schl*‐GWR3R2 vector to generate the pTKan‐*pC4H::schl*::*qsuB* construct, which was introduced into wild‐type *Arabidopsis* plants (ecotype Col‐0) via *Agrobacterium*‐mediated transformation (Bechtold and Pelletier, 1998).

### Western blot analysis

Proteins from *Arabidopsis* stems were extracted using a buffer containing 250 mm Tris‐HCl pH 8.5, 25 mm EDTA, 2 mm DTT, 5 mm β‐mercaptoethanol and 10% sucrose, and were quantified using the Bradford method (Bradford, 1976). Proteins (15 μg) were separated by SDS‐PAGE, blotted and immunodetected using a universal antibody, as previously described (Eudes *et al*., 2011).

### Methanol‐soluble metabolites extraction

*Arabidopsis* stems of 5‐week‐old wild‐type and T3 homozygous *C4H::qsuB* lines were collected in liquid nitrogen and stored at −80 °C until further utilization. Prior the metabolite extraction, collected stems were pulverized in liquid nitrogen. For extraction of methanol‐soluble metabolites, 700–1000 mg of frozen stem powder was mixed with 2 mL of 80% (v/v) methanol–water and mixed (1400 rpm) for 15 min at 70 °C. This step was repeated four times. Pooled extracts were cleared by centrifugation (5 min, 20 000 ***g**,* at room temperature), mixed with 4 mL of analytical grade water and filtered using Amicon Ultra centrifugal filters (10 000 Da MW cut‐off regenerated cellulose membrane; EMD Millipore, Billerica, MA, USA). Filtered extracts were lyophilized and the resulting pellets dissolved in 200 μL 50% (v/v) methanol–water prior to LC‐MS analysis. An acid hydrolysis of the samples was performed for the quantification of protocatechuate and salicylate; an aliquot of the filtered extracts was dried under vacuum, resuspended with 1 N HCl and incubated at 95 °C for 3 h. The mixture was subjected to three ethyl acetate partitioning steps. Ethyl acetate fractions were pooled, dried in vacuo and resuspended in 50% (v/v) methanol–water prior to LC‐MS analysis.

### LC‐MS analysis

Phenolic acids, phenolic aldehydes, and aromatic amino acids were analyzed using high‐performance liquid chromatography (HPLC), electrospray ionization (ESI), and time‐of‐flight (TOF) mass spectrometry (MS) as previously described in Eudes *et al*. (2013) and Bokinsky *et al*. (2013), respectively. Aromatic alcohols were analysed by HPLC – atmospheric pressure chemical ionization (APCI) – TOF MS. Their separation was conducted on an Agilent 1200 Series Rapid Resolution HPLC system (Agilent Technologies Inc., Santa Clara, CA, USA) using a Phenomenex Kinetex XB‐C18 (100 mm length, 2.1 mm internal diameter and 2.6 μm particle size; Phenomenex, Torrance, CA, USA). The mobile phase was composed of 0.1% formic acid in water (solvent A) and methanol (solvent B). The elution gradient was as follows: from 5% B to 25% B for 6 min, 25% B to 5% B for 1 min and held at 5% B for a further 3 min. A flow rate of 0.5 mL/min was used throughout. The column compartment and sample tray were set to 50 and 4 °C, respectively. The HPLC system was coupled to an Agilent Technologies 6210 LC/TOF mass spectrometer with a 1:4 postcolumn split. Mass spectrometric detection was conducted using APCI in the positive ion mode. MS experiments were carried out in the full‐scan mode, at 0.86 spectra/second, for the detection of [M–H_2_O+H]^+^ ions. Drying and nebulizing gases were set to 10 L/min and 25 psi, respectively, and a drying gas temperature of 330 °C was used throughout. The vaporizer and corona were set to 350 °C and 4 μA, respectively, and a capillary voltage of 3500 V was also used. Fragmentor and OCT 1 RF voltages were each set to 135 V, while the skimmer voltage was set to 50 V. Data acquisition and processing were performed by the MassHunter software package (Agilent Technologies Inc.). Metabolites were quantified via 10‐point calibration curves of authentic standard compounds for which the *R* ^2^ coefficients were ≥0.99. Representative LC‐MS chromatograms obtained from solutions of standard compounds and from plant metabolite extracts are illustrated in Figure S7.

### Lignin content and composition

The biomass from senesced wild‐type plants and T3 homozygous *C4H::qsuB* lines was used to determine lignin content and composition. Biomass was extracted sequentially by sonication (20 min) with 80% ethanol (three times), acetone (one time), chloroform–methanol (1:1, v/v, one time) and acetone (one time). The standard NREL biomass protocol was used to measure lignin content (Sluiter *et al*., 2008). The chemical composition of lignin was analysed by pyrolysis‐gas chromatography (GC)/mass spectrometry (MS) using a previously described method with some modifications (Del Río *et al*., 2012). Pyrolysis of biomass was performed with a Pyroprobe 5200 (CDS Analytical Inc., Oxford, PA, USA) connected with GC/MS (Thermo Electron Corporation with Trace GC Ultra and Polaris‐Q MS) equipped with an Agilent HP‐5MS column (30 m × 0.25 mm i.d., 0.25 μm film thickness). The pyrolysis was carried out at 550 °C. The chromatograph was programmed from 50 °C (1 min) to 300 °C at a rate of 30 °C/min; the final temperature was held for 10 min. Helium was used as the carrier gas at a constant flow rate of 1 mL/min. The mass spectrometer was operated in scan mode and the ion source was maintained at 300 °C. The compounds were identified by comparing their mass spectra with those of the NIST library and those previously reported (Del Río and Gutiérrez, 2006; Ralph and Hatfield, 1991). Peak molar areas were calculated for the lignin degradation products, and the summed areas were normalized.

### Cell wall‐bound aromatics extraction

The biomass from senesced wild‐type plants and T3 homozygous *C4H::qsuB* lines was used to measure cell wall‐bound aromatics. Extracted biomass (10 mg) was mixed with 500 μL of 2 m NaOH and shaken at 1400 rpm for 24 h at 30 °C. The mixture was acidified with 100 μL of concentrated HCl and subjected to three ethyl acetate partitioning steps. Ethyl acetate fractions were pooled, dried in vacuo and suspended in 50% (v/v) methanol–water prior to LC‐MS analysis.

### 2D ^13^C‐^1^H heteronuclear single‐quantum coherence (HSQC) NMR spectroscopy

Stem material from wild‐type and *pC4H::schl::qsuB‐1* plants was extracted and ball‐milled as previously described (Kim and Ralph, 2010; Mansfield *et al*., 2012). The gels were formed using DMSO‐d_6_/pyridine‐d_5_ (4:1) and sonicated until homogenous in a Branson 2510 table‐top cleaner (Branson Ultrasonic Corporation, Danbury, CT, USA). The temperature of the bath was closely monitored and maintained below 55 °C. The homogeneous solutions were transferred to NMR tubes. HSQC spectra were acquired at 25 °C using a Bruker Avance‐600 MHz instrument equipped with a 5 mm inverse‐gradient ^1^H/^13^C cryoprobe using a hsqcetgpsisp2.2 pulse programme (ns = 400, ds = 16, number of increments = 256, d_1_ = 1.0 s) (Heikkinen *et al*., 2003). Chemical shifts were referenced to the central DMSO peak (δ_C_/δ_H_ 39.5/2.5 ppm). Assignment of the HSQC spectra was described elsewhere (Kim and Ralph, 2010; Yelle *et al*., 2008). A semi‐quantitative analysis of the volume integrals of the HSQC correlation peaks was performed using Bruker's Topspin 3.1 (Windows) processing software. A Gaussian apodization in F_2_ (LB = −0.50, GB = 0.001) and squared cosine‐bell in F_1_ (LB = −0.10, GB = 0.001) were applied prior to 2D Fourier transformation.

### Isolation of cellulolytic enzyme lignin

Stem material from wild‐type and *pC4H::schl::qsuB‐1* plants was extracted and ball‐milled for 3 h per 500 mg of sample (in 10 min on/10 min off cycles) using a PM100 ball mill (Retsch, Newtown, PA, USA) vibrating at 600 rpm in zirconium dioxide vessels (50 mL) containing ZrO_2_ ball bearings (10 × 10 mm). Ball‐milled walls were digested four times over 3 days at 50 °C with the polysaccharidases Cellic CTec2 and HTec2 (Novozymes, Davis, CA, USA) and pectinase from *Aspergillus niger* (Sigma‐Aldrich, St. Louis, MO, USA) in sodium citrate buffer (pH 5.0). The obtained cellulolytic lignin was washed with deionized water and lyophilized overnight.

### Size‐exclusion chromatography

Lignin solutions, 1% (w/v), were prepared in analytical grade 1‐methyl‐2‐pyrrolidinone (NMP). The polydispersity of dissolved lignin was determined using analytical techniques involving SEC UV‐F_250/400_ as previously described (George *et al*., 2011). An Agilent 1200 series binary LC system (G1312B) equipped with diode‐array (G1315D) and fluorescence (G1321A) detectors was used. Separation was achieved with a Mixed‐D column (5 μm particle size, 300 mm × 7.5 mm i.d., linear molecular mass range of 200 to 400 000 u, Agilent Technologies Inc.) at 80 °C using a mobile phase of NMP at a flow rate of 0.5 mL/min. Absorbance of materials eluting from the column was detected using UV‐F fluorescence (Ex_250_/Em_450_). Spectral intensities were area‐normalized, and molecular mass estimates were determined after calibration of the system with polystyrene standards.

### Cell wall pretreatments and saccharification

Ball‐milled senesced stems (10 mg) were mixed with 340 μL of water, 340 μL of H_2_SO_4_ (1.2%, w/v) or 340 μL of NaOH (0.25%, w/v) for hot water, dilute acid or dilute alkali pretreatments, respectively, shaken at 1400 rpm (30 °C, 30 min) and autoclaved at 120 °C for 1 h. Samples pretreated with dilute acid were neutralized with 5 N NaOH (25 μL). Saccharification was initiated by adding 650 μL of 100 mm sodium citrate buffer pH 5 (for hot water‐ and dilute alkali‐pretreated samples) or 625 μL of 80 mm sodium citrate buffer pH 6.2 (for dilute acid‐pretreated samples) containing 80 μg/mL tetracycline and 1% w/w or 0.2% w/w Cellic CTec2 cellulase (Novozymes). After 72 h of incubation at 50 °C with shaking (800 rpm), samples were centrifuged (20 000 ***g***, 3 min) and 10 μL of the supernatant was collected for measurement of reducing sugars using the 3,5‐dinitrosalicylic acid assay and glucose solutions as standards (Miller, 1959).

## Conflict of interests

JDK has financial conflict of interests in Amyris, LS9 and Lygos. DL has financial conflict of interests in Afingen.

## Supporting information


**Figure S1** Subcellular localization of SCHL::QsuB.
**Figure S2** Summary of the fold changes observed for the methanol‐soluble metabolites extracted from plants expressing QsuB.
**Figure S3** Partial short‐range ^13^C–^1^H (HSQC) spectra (aliphatic region) of cell wall material from mature senesced stems of wild‐type and *pC4H::schl::qsuB‐1* plants.
**Figure S4** Lignin staining by phloroglucinol‐HCl of stem sections from 5‐week‐old wild‐type and *pC4H::schl::qsuB* plants.
**Figure S5** Picture of 12‐week‐old wild‐type (WT) and *pC4H::schl::qsuB* (*C4H::qsuB*) plants.
**Figure S6** Detection by RT‐PCR of HCT, C3H and CSE transcripts using stem mRNA from 5‐week‐old wild‐type (WT) and *pC4H::schl::qsuB* (*C4H::qsuB*) plants. Two plants per line were analysed (#1 and #2). *Tub8*‐specific primers were used to assess cDNA quality for each sample.
**Figure S7** Representative LC‐MS chromatograms obtained from solutions of standard compounds and from metabolite (methanol‐soluble or cell wall‐bound) extracts from wild‐type (WT) and/or *pC4H::schl::qsuB* (*C4H::qsuB*) plants.
**Table S1** Characteristics and relative molar abundances (%) of the compounds released after Pyro‐GC/MS of extractive‐free senesced mature stems from wild‐type (WT) and *pC4H::schl::qsuB* (*C4H::qsuB*) plants. Values in brackets are the SE from duplicate analyses.
**Data S1** Supporting experimental procedures for supplemental data.
