# Supplementary material for: Expression of a bacterial 3‐dehydroshikimate dehydratase reduces lignin content and improves biomass saccharification efficiency
Source: Plant Biotechnol J. 2015 Jan 13;13(9):1241–50. doi: 10.1111/pbi.12310 (PMC6680230; doi:10.1111/pbi.12310)
Supplement: Supplementary file 1 — Figure S1 Subcellular localization of SCHL::QsuB. Figure S2 Summary of the fold changes observed for the methanol‐soluble metabolites extracted from plants expressing QsuB. Figure S3 Partial short‐range 13C–1H (HSQC) spectra (aliphatic region) of cell wall material from mature senesced stems of wild‐type and pC4H::schl::qsuB‐1 plants. Figure S4 Lignin staining by phloroglucinol‐HCl of stem sections from 5‐week‐old wild‐type and pC4H::schl::qsuB plants. Figure S5 Picture of 12‐week‐old wild‐type (WT) and pC4H::schl::qsuB (C4H::qsuB) plants. Figure S6 Detection by RT‐PCR of HCT, C3H and CSE transcripts using stem mRNA from 5‐week‐old wild‐type (WT) and pC4H::schl::qsuB (C4H::qsuB) plants. Two plants per line were analysed (#1 and #2). Tub8‐specific primers were used to assess cDNA quality for each sample. Figure S7 Representative LC‐MS chromatograms obtained from solutions of standard compounds and from metabolite (methanol‐soluble or cell wall‐bound) extracts from wild‐type (WT) and/or pC4H::schl::qsuB (C4H::qsuB) plants. Table S1 Characteristics and relative molar abundances (%) of the compounds released after Pyro‐GC/MS of extractive‐free senesced mature stems from wild‐type (WT) and pC4H::schl::qsuB (C4H::qsuB) plants. Values in brackets are the SE from duplicate analyses. Data S1 Supporting experimental procedures for supplemental data. [file PBI-13-1241-s001.pdf]

Table S1. Characteristics and relative molar abundances (%) of the compounds released after pyro-GC/MS of extractive-free senesced mature stems from wild-type (WT) and *pC4H::schl::qsuB* (*C4H::qsuB*) plants. Values in brackets are the SE from three biological replicates (*n* = 3).

Compound name	Origin	Formula	Molecular mass	Main mass fragments	Elution time (min)	WT (%)	*C4H::qsuB-1* (%)	*C4H::qsuB-3* (%)	*C4H::qsuB-6* (%)	*C4H::qsuB-7* (%)
Phenol	H	C_6H_6O	94	65, 66, *94*	4.28	0.5 (0.1)	3.5 (0.6)	2.1 (0.1)	5.0 (0.7)	3.0 (0.5)
2-Methylphenol	H	C_7H_8O	108	77, *107*, 108	4.96	0.5 (0.1)	1.5 (0.3)	1.1 (0.1)	1.8 (0.1)	1.3 (0.1)
3-Methylphenol	H	C_7H_8O	108	77, *107*, 108	5.16	1.3 (0.1)	6.3 (0.2)	3.5 (0.4)	7.1 (0.6)	4.5 (0.8)
2-Methoxyphenol	G	$C_7H_8O_2$	124	81, *109*, 124	5.34	8.0 (0.9)	5.3 (0.6)	4.6 (0.2)	4.4 (0.2)	5.2 (0.6)
2,5-Dimethylphenol	H	$C_8H_{10}O$	122	77, 107, *122*	5.93	0.6 (0.0)	2.5 (0.4)	1.9 (0.2)	2.7 (0.2)	1.9 (0.1)
4-Ethylphenol	H	$C_8H_{10}O$	122	77, *107*, 122	6.15	0.4 (0.1)	1.7 (0.1)	2.1 (0.1)	3.5 (0.5)	2.2 (0.3)
2-Methoxy-5-methylphenol	G	$C_8H_{10}O_2$	138	95, *123*, 138	6.45	6.2 (0.5)	5.6 (0.8)	4.2 (0.3)	5.0 (0.5)	4.3 (0.3)
4-Ethyl-2-methoxyphenol	G	$C_9H_{12}O_2$	152	122, *137*, 152	7.45	2.4 (0.3)	2.2 (0.4)	1.9 (0.0)	1.5 (0.3)	2.2 (0.3)
4-Ethenyl-2-methoxyphenol	G	$C_9H_{10}O_2$	150	107, *135*, 150	7.88	18.1 (1.4)	12.5 (0.6)	12.8 (0.7)	13.2 (1.4)	13.9 (0.4)
2,6-Dimethoxyphenol	S	$C_8H_{10}O_3$	154	111, 139, *154*	8.36	4.5 (0.3)	5.6 (0.4)	6.0 (0.2)	4.9 (0.6)	5.6 (0.3)
2-Methoxy-4-propenylphenol	G	$C_{10}H_{12}O_2$	164	131, 149, *164*	8.41	2.5 (0.1)	1.5 (0.2)	1.8 (0.2)	1.6 (0.2)	1.5 (0.1)
4-hydroxy-3-methoxyphenylacetaldehyde	G	$C_{10}H_{14}O_2$	166	122, *137*, 166	8.52	0.5 (0.0)	0.2 (0.1)	0.4 (0.0)	0.3 (0.1)	0.4 (0.0)
4-Hydroxy-3-methoxybenzaldehyde	G	C8H8O3	152	109, 151, *152*	9.02	3.2 (0.5)	1.7 (0.2)	2.3 (0.1)	1.1 (0.3)	2.9 (0.1)
4-Methyl-2,6-dimethoxyphenol	S	$C_9H_{12}O_2$	168	125, 153, *168*	9.47	2.4 (0.1)	2.8 (0.5)	2.5 (0.4)	2.2 (0.6)	2.9 (0.5)
2-Methoxy-4-propenylphenol	G	$C_{10}H_{12}O_2$	164	131, 149, *164*	9.52	11.0 (0.2)	6.3 (0.3)	7.1 (0.5)	5.5 (1.5)	7.0 (0.6)
4-Ethyl-2,6-dimethoxyphenol	S	$C_{10}H_{14}O_3$	182	*167*, 182	10.42	1.6 (0.1)	3.9 (0.3)	2.8 (0.1)	2.3 (0.2)	2.6 (0.2)
4-Hydroxy-3-methoxyphenyl acetone	G	$C_{10}H_{12}O_3$	180	122, *137*, 180	10.56	2.0 (0.1)	2.0 (0.4)	2.2 (0.2)	2.3 (0.2)	2.2 (0.2)
4-Hydroxy-3,5-dimethoxystyrene	S	$C_{10}H_{12}O_3$	180	137, 165, *180*	10.88	11.3 (1.2)	17.3 (0.6)	19.9 (0.8)	16.4 (1.5)	15.5 (0.8)
4-Allyl-2,6-dimetoxyphenol	S	$C_{11}H_{14}O_3$	194	167, 179, *194*	11.32	1.5 (0.1)	1.5 (0.2)	2.1 (0.2)	1.6 (0.1)	1.9 (0.1)
4-Hydroxy-3,5-dimethoxybenzaldehyde	S	$C_9H_{10}O_4$	182	167, 181, *182*	12.07	0.9 (0.3)	1.9 (0.1)	2.0 (0.2)	2.5 (0.6)	2.2 (0.1)
4-Propinyl-2,6-dimethoxyphenol	S	$C_{11}H_{12}O_3$	192	106, 131, 177, *192*	12.23	0.4 (0.2)	0.5 (0.2)	0.5 (0.0)	0.4 (0.1)	0.5 (0.1)
4-Propenyl-2,6-dimethoxyphenol	S	$C_{11}H_{14}O_3$	194	167, 179, *194*	12.43	7.5 (0.6)	8.5 (0.4)	9.7 (0.2)	8.4 (1.2)	8.4 (0.5)
4-Hydroxy-3,5-dimethoxyacetophenone	S	$C_{10}H_{12}O_4$	196	153, *181*, 196	12.88	0.8 (0.0)	0.6 (0.2)	1.4 (0.1)	1.1 (0.4)	0.9 (0.1)
4-hydroxy-3-methoxycinnamaldehyde	G	$C_{10}H_{10}O_3$	178	107, 135, 147, *178*	13.00	10.2 (0.7)	1.4 (0.3)	2.1 (0.3)	1.9 (0.3)	4.2 (1.1)
4-hydroxy-3,5-dimethoxyphenylacetone	S	$C_{11}H_{14}O_4$	210	123, *167*, 210	13.26	1.5 (0.2)	2.8 (0.4)	2.8 (0.1)	3.0 (0.3)	2.6 (0.2)
4-Hydroxy-3,5-dimethoxyphenylethanone	S	$C_{10}H_{12}O_4$	196	153, *181*, 196	13.83	0.2 (0.0)	0.3 (0.0)	0.2 (0.1)	0.3 (0.1)	0.2 (0.0)
% H-units						3.3 (0.2)	15.5 (0.2)	10.8 (0.4)	20.0 (1.0)	12.8 (1.8)
% G-units						64.1 (1.9)	38.9 (0.6)	39.4 (1.2)	36.9 (2.8)	43.8 (1.3)
% S-units						32.6 (2.0)	45.6 (0.5)	49.8 (0.9)	43.1 (3.5)	43.4 (1.9)

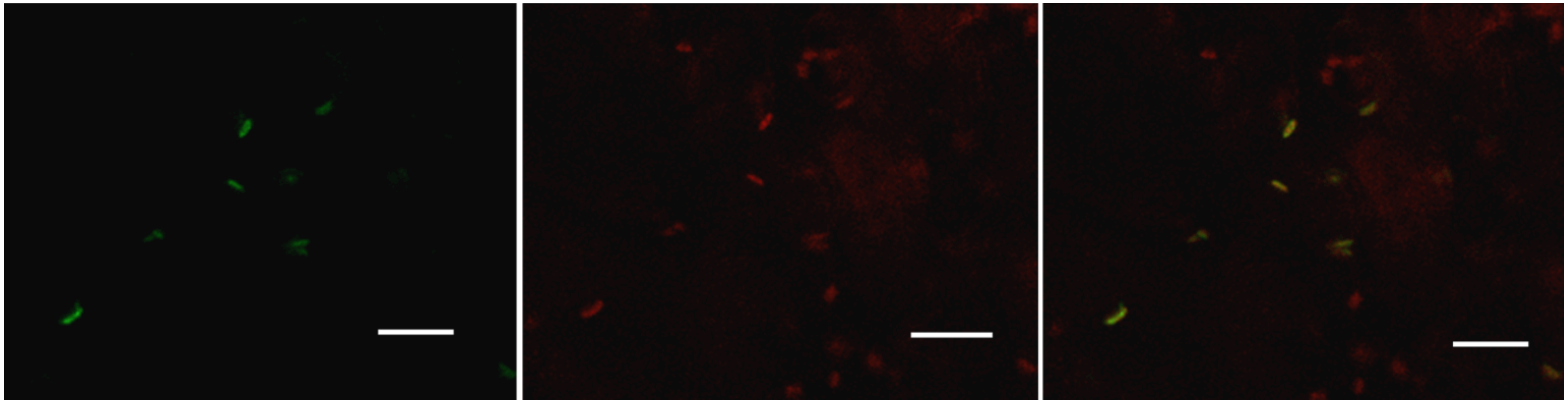

Supplemental Figure S1: Subcellular localization of SCHL::QsuB.
The left panel displays the transient expression of SCHL::QsuB::YFP fusion protein expressed under the control of the 35S promoter in epidermal cells of *N. benthamiana* and imaged by confocal laser scanning microscopy. The central panel displays fluorescing chloroplasts and the right panel shows the merged images (colocalizations are visible as yellow dots). Scale bars = 20 μm.

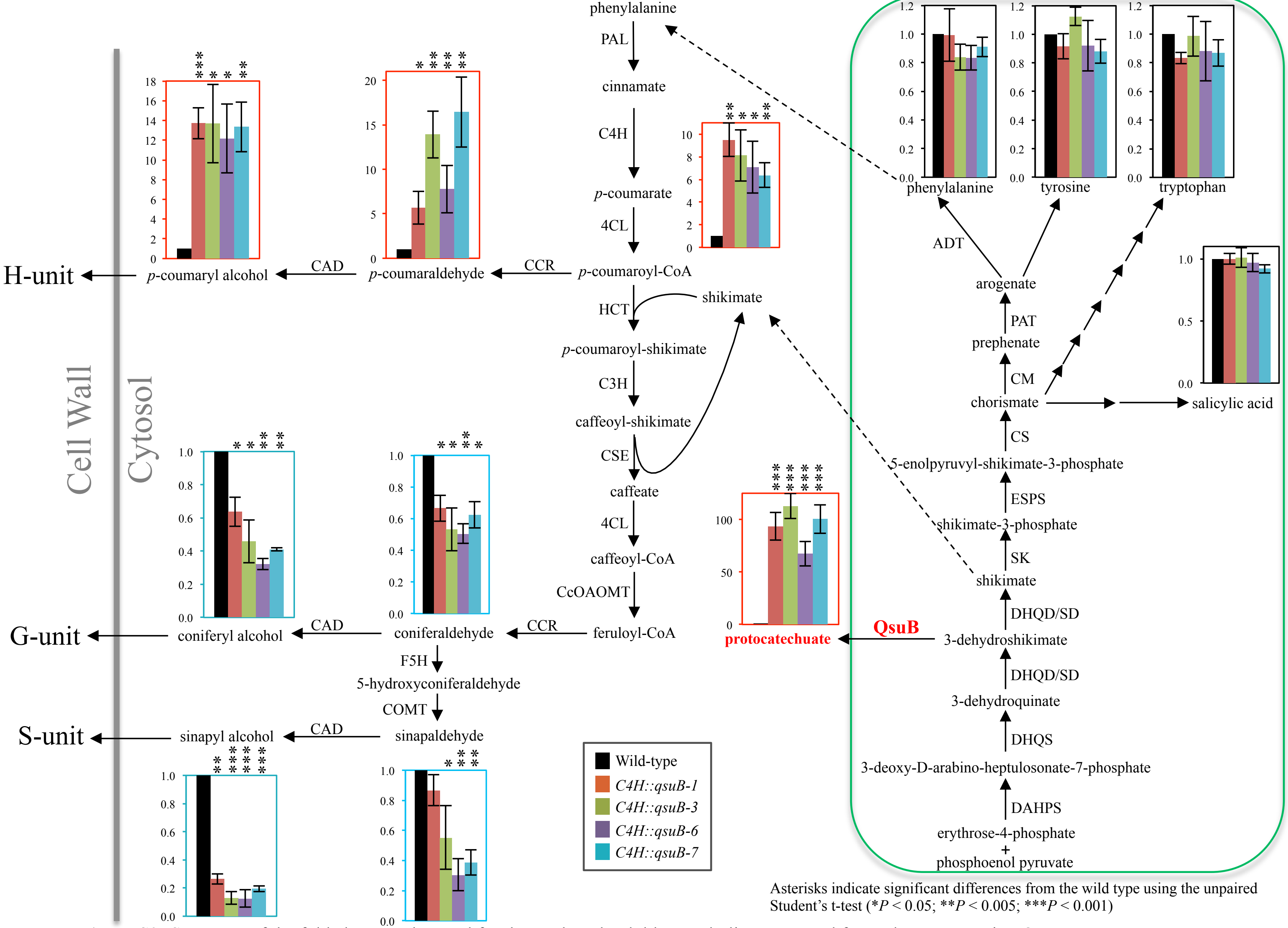

Asterisks indicate significant differences from the wild type using the unpaired Student's t-test (*$P < 0.05$; **$P < 0.005$; ***$P < 0.001$)

Supplemental Figure S2: Summary of the fold changes observed for the methanol-soluble metabolites extracted from plants expressing QsuB.

Abbreviations: DAHPS, 3-deoxy-D-arabino-heptulosonate 7-phosphate synthase; DHQS, 3-dehydroquinate synthase; DHQD/SD, 3-dehydroquinate dehydratase; SK, shikimate kinase; ESPS, 3-phosphoshikimate 1-carboxyvinyltransferase; CS, chorismate synthase; CM, chorismate mutase; PAT, prephenate aminotransferase; ADT, arogenate dehydratase; PAL, phenylalanine ammonia-lyase; C4H, cinnamate 4-hydroxylase; CSE, caffeoyl shikimate esterase; 4CL, 4-coumarate CoA ligase; CAD, cinnamyl alcohol dehydrogenase; F5H, ferulate 5-hydroxylase; C3H, coumarate 3-hydroxylase; COMT, caffeic acid 3-O-methyltransferase; CCR, cinnamoyl-CoA reductase; HCT, hydroxycinnamoyl-Coenzyme A shikimate/quinate hydroxycinnamoyltransferase; CCoAOMT, caffeoyl/CoA-3-*O*-methyltransferase; qsuB, 3-dehydroshikimate dehydratase from *Corynebacterium glutamicum*.

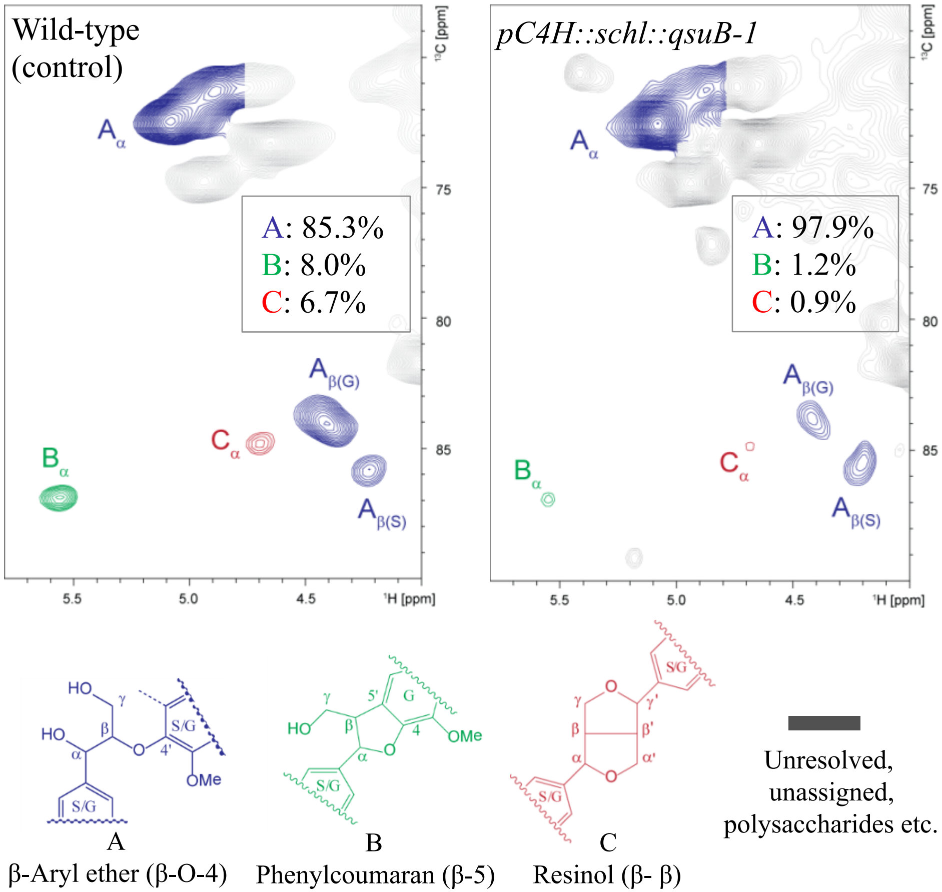

Supplemental Figure S3: Partial short-range ^{13}C–^{1}H (HSQC) spectra (aliphatic region) of cell wall material from mature senesced stems of wild-type and *pC4H::schl::qsuB-1* plants. Integration values for the α-C/H correlation peaks from the major lignin interunit structures A-C are provided.

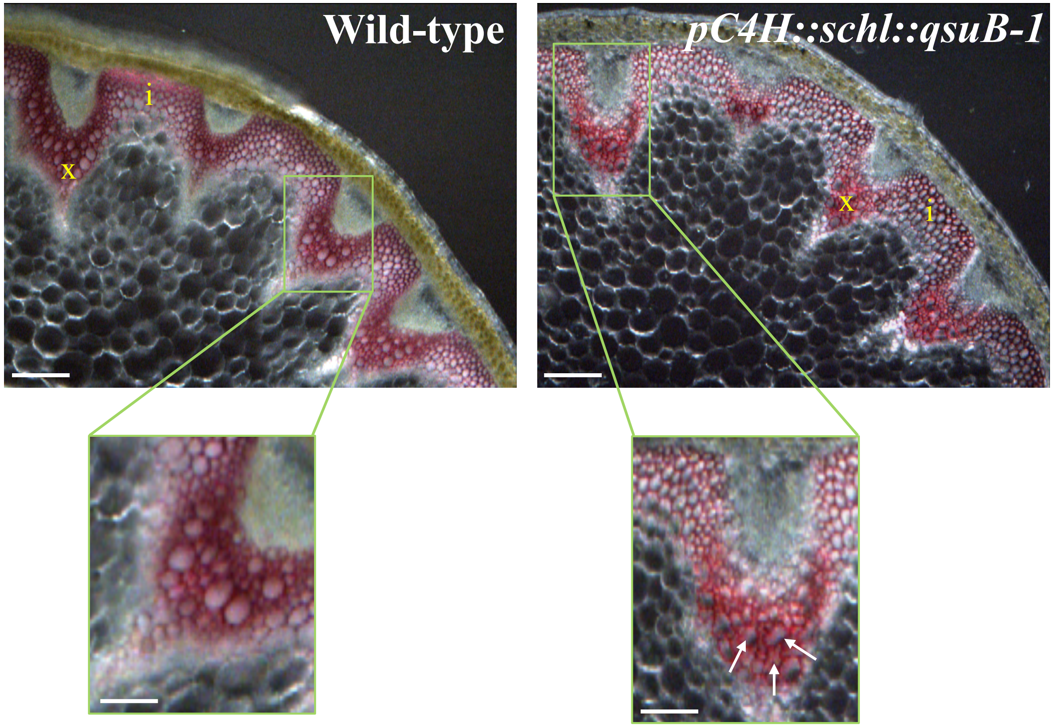

Supplemental Figure S4: Lignin staining by phloroglucinol-HCl of stem sections from 5-week-old wild-type and *pC4H::schl::qsuB-1* plants.
i, interfascicular fibers; x, xylem. Bars represent 50 µm (top panels) and 20 µm (bottom panels).
Note the collapsed xylem vessels (white arrows) observed for the *pC4H::schl::qsuB-1* line.

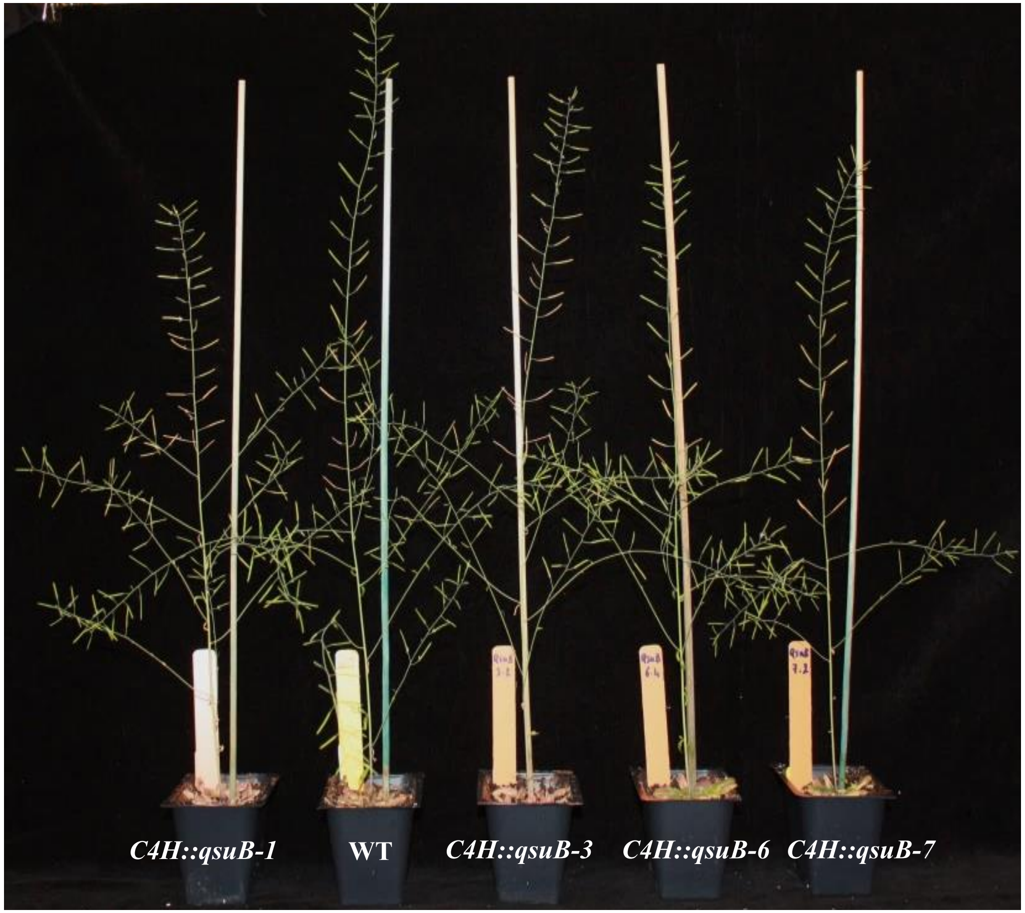

Supplemental Figure S5. Picture of 12-week-old wild-type (WT) and *pC4H::schl::qsuB* (*C4H::qsuB*) plants.

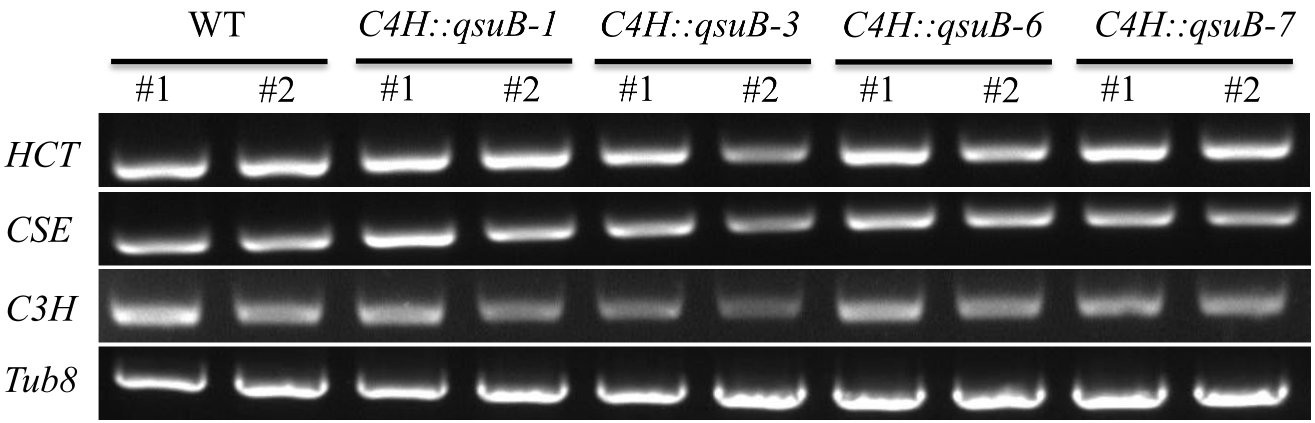

Supplemental Figure S6: Detection by RT-PCR of *HCT*, *C3H* and *CSE* transcripts using stem mRNA from 5-week-old wild-type (WT) and *pC4H::schl::qsuB* (*C4H::qsuB*) plants. Two plants per line were analyzed (#1 and #2). *Tub8*-specific primers were used to assess cDNA quality for each sample.

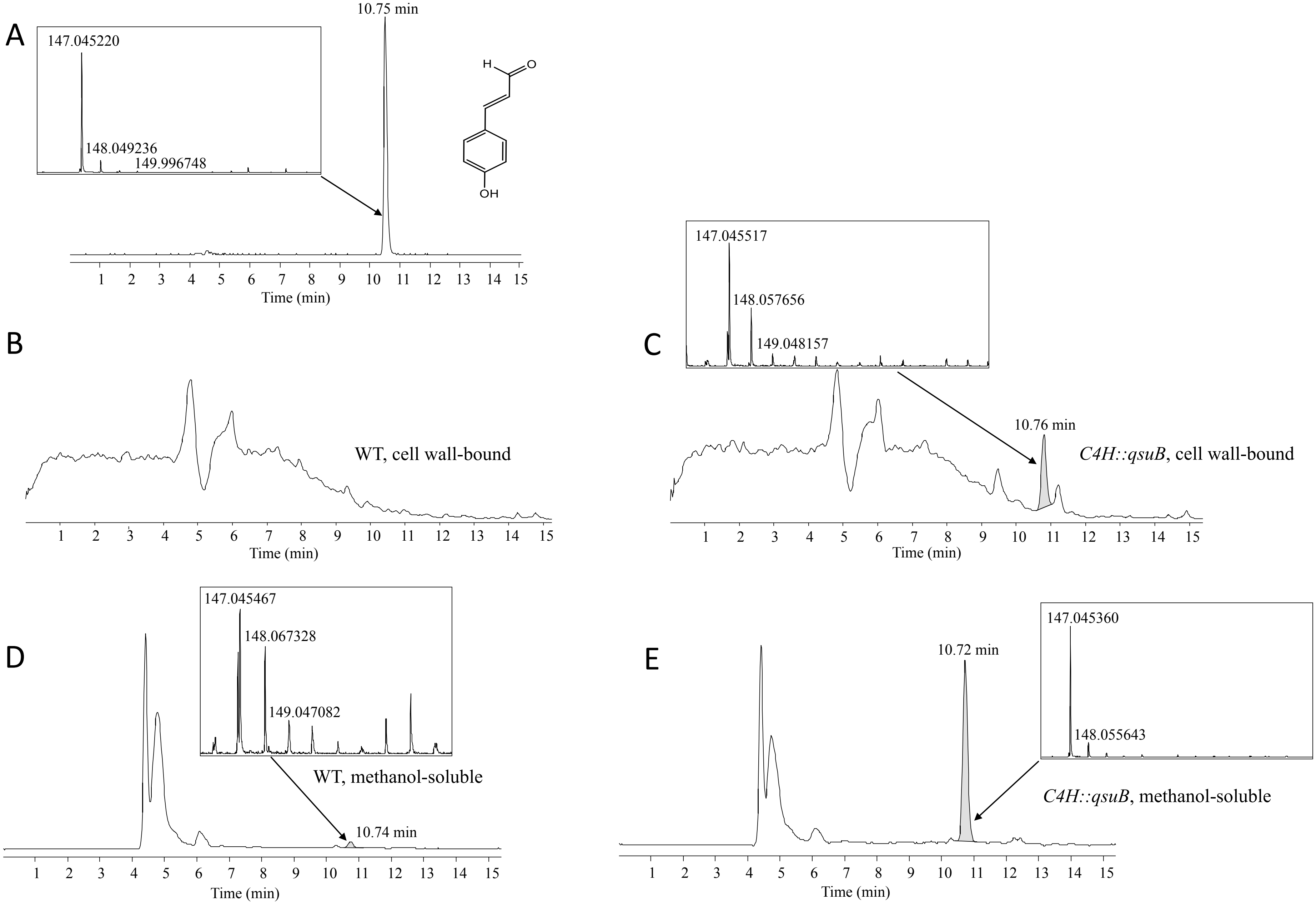

Supplemental Figure S7. Representative LC-MS chromatograms obtained from solutions of standard compounds (A, F, H, J, L, N and P) and from metabolite (methanol-soluble or cell wall-bound) extracts from wild-type (WT) and/or *pC4H::schl::qsuB* (*C4H::qsuB*) plants.
A-E: *p*-coumaraldehyde, F-G: coniferaldehyde, H-I: sinapaldehyde, J-K: *p*-coumarate, L-M: *p*-coumaroyl alcohol, N-O: coniferyl alcohol, P-Q: sinapyl alcohol.

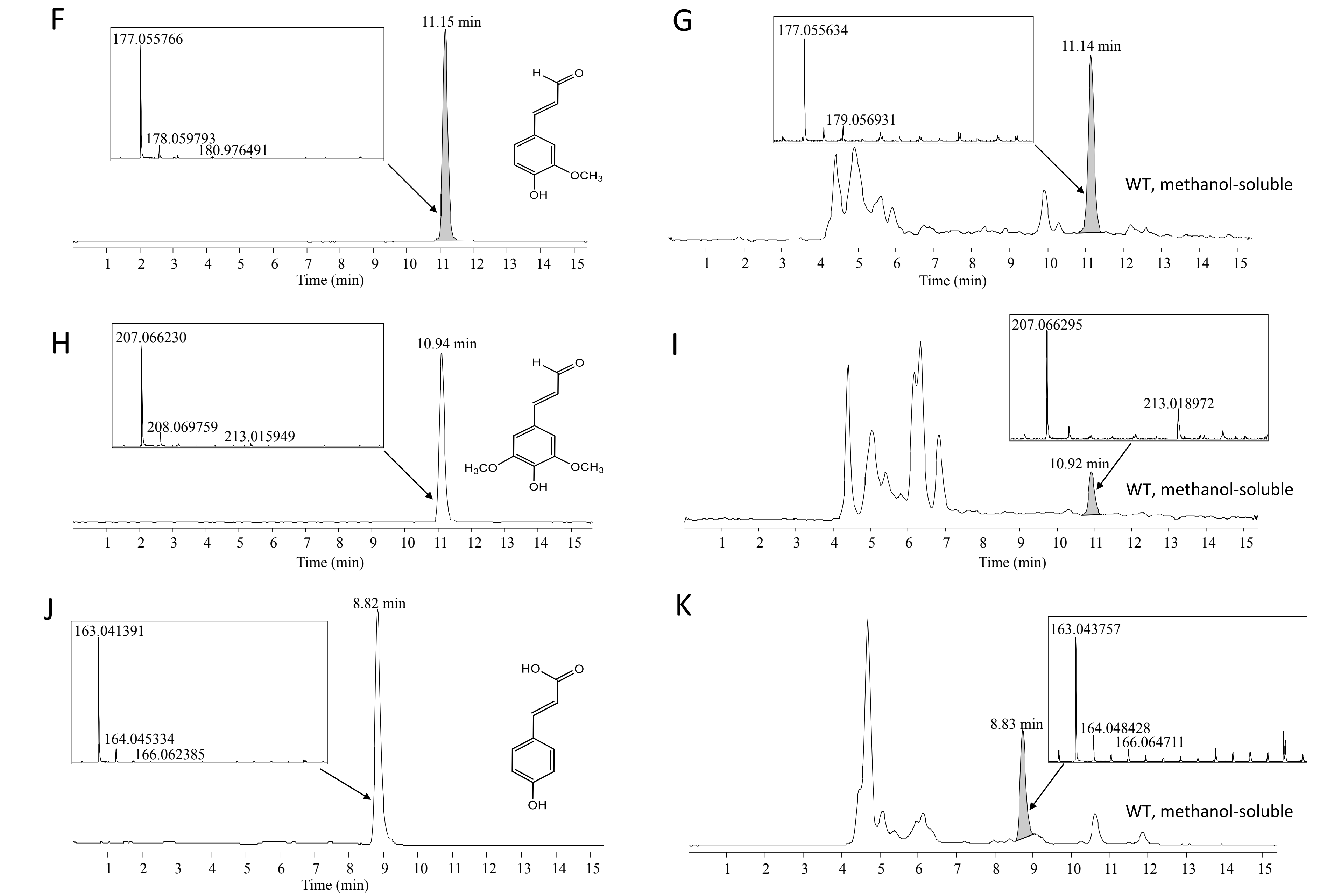

Supplemental Figure S7. Representative LC-MS chromatograms obtained from solutions of standard compounds (A, F, H, J, L, N and P) and from metabolite (methanol-soluble or cell wall-bound) extracts from wild-type (WT) and/or *pC4H::schl::qsuB* (*C4H::qsuB*) plants.
A-E: *p*-coumaraldehyde, F-G: coniferaldehyde, H-I: sinapaldehyde, J-K: *p*-coumarate, L-M: *p*-coumaroyl alcohol, N-O: coniferyl alcohol, P-Q: sinapyl alcohol.

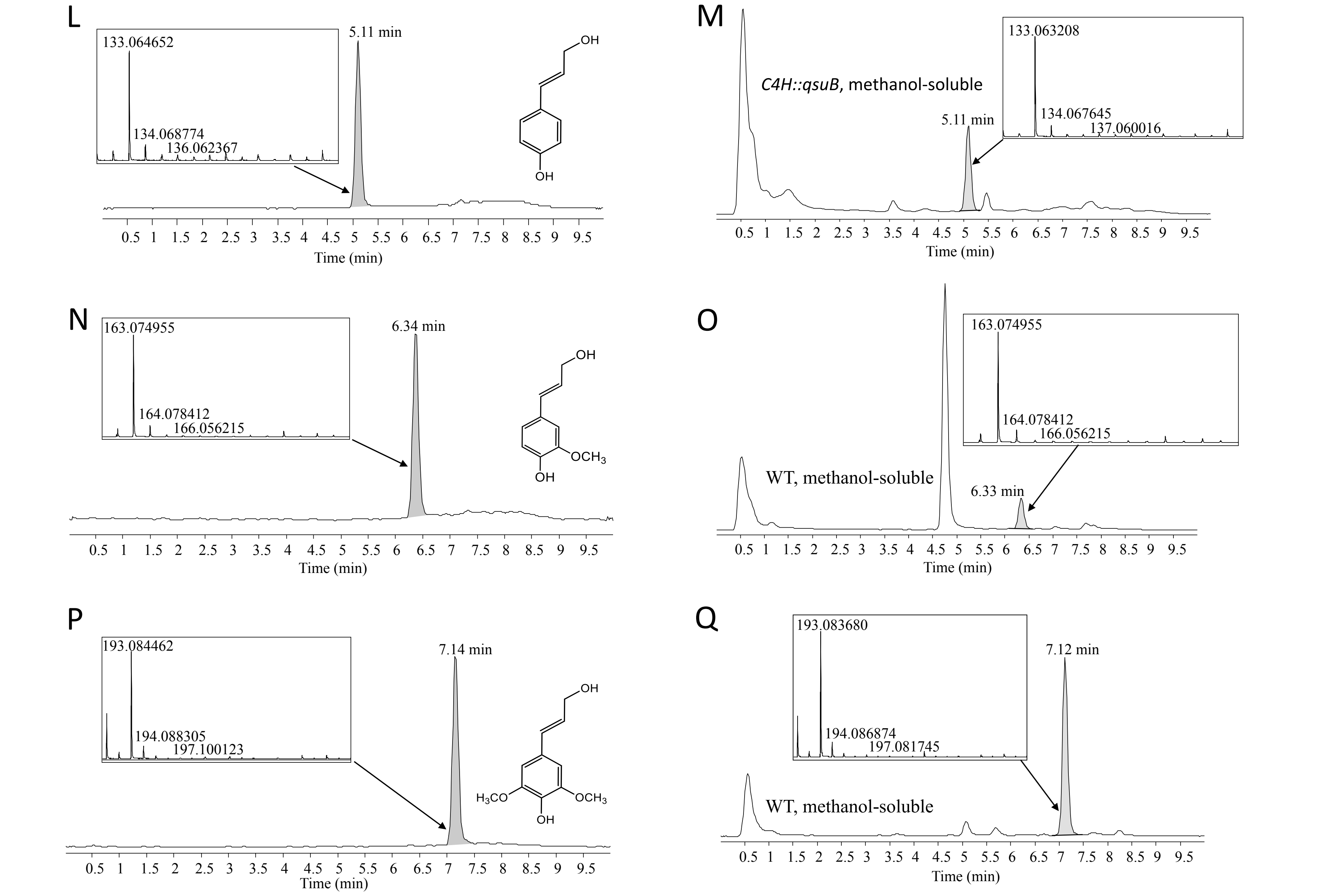

Supplemental Figure S7. Representative LC-MS chromatograms obtained from solutions of standard compounds (A, F, H, J, L, N and P) and from metabolite (methanol-soluble or cell wall-bound) extracts from wild-type (WT) and/or *pC4H::schl::qsuB* (*C4H::qsuB*) plants.
A-E: *p*-coumaraldehyde, F-G: coniferaldehyde, H-I: sinapaldehyde, J-K: *p*-coumarate, L-M: *p*-coumaroyl alcohol, N-O: coniferyl alcohol, P-Q: sinapyl alcohol.

Experimental Procedures for Supplemental Data

Subcellular localization of QsuB

The *schl-qsuB* nucleotide sequence from the pTkan-*pC4H::schl-qsuB* construct was amplified using oligonucleotides 5'-GGGGACAAGTTTGTACAAAAAAGCAGGCTTCATGGCTTCGATCTCCTCCT-3' (attB1 site underlined) and 5'-GGGGACCACTTTGTACAAGAAAGCTGGGTCGTTTGGGATACCTCTCTCTAAATCTC-3' (attB2 site underlined) and cloned into the Gateway pDONR221-f1 entry vector (Lalonde *et al*., 2010). A sequence-verified entry clone was LR recombined with the pTKan-*p35S*-GWR1R2-YFP vector to generate the pTKan-*p35S-schl-qsuB-YFP* construct. Infiltration of four-week-old *N. benthamiana* leaves was done using the *Agrobacterium* strain GV3101, following the method described by Sparkes *et al*. (2006). Plants transiently expressing the SCHL-QsuB-YFP fusion protein were analyzed by confocal laser scanning microscopy two days after the infiltration. The microscopy was performed using a Zeiss LSM 710 device (Carl Zeiss Microscopy, Jena, Germany) equipped with an argon laser (excitation at 514 nm and emission collected at 510 to 545 nm).

Lignin histochemical staining

Histochemical staining was performed as described by Pradhan-Mitra and Loque (2014). Basal stem transverse sections (100 µm-thick) were obtained using a vibratome. Sections were incubated for 3 min in phloroglucinol-HCl reagent (VWR International, Brisbane, CA), rinsed with water, and observed using bright field light microscopy (Leica Microsystems Inc., Buffalo Grove, IL).

RNA extraction and RT-PCR

Total RNA (1 μg) were extracted using the Plant RNeasy extraction kit (Qiagen, Valencia, CA) and reverse-transcribed using the Transcriptor First Strand cDNA Synthesis Kit (Roche Applied Science, Indianapolis, IN). The obtained cDNA preparations were quality-controlled and calibrated for semi-quantitative PCR using *tub8*-specific oligonucleotides (5´-GGGCTAAAGGACACTACACTG-3´ and 5´-CCTCCTGCACTTCCACTTCGTCTTC-3´). The detection of *HCT*, *C3H* and *CSE* transcripts was made using the following oligonucleotides: 5´-GGCAGGGCATGTGTGGAGATC-3´ / 5´-TCTCTTGTAAGCCTGGAGTCC-3´ (*HCT*), 5´-GGTCTTCTATGGGATATGATCACGGC-3´ / 5´-CGAAGGCAACCGAGGCGTTGC-3´ (*C3H*), and 5´-GGTGAATCGATGGGAGGTCTTGTGAC-3´ / 5´-CTATCTCAGCGTTCTCGTCAGGCTCTC -3´ (*CSE*).

References

Lalonde, S., Sero, A., Pratelli, R., Pilot, G., Chen, J., Sardi, M.I., Parsa, S.A., Kim, D.-Y., Acharya, B.R., Stein, E.V., Hu, H.-C., Villiers, F., Takeda, K., Yang, Y., Han, Y.S., Schwacke, R., Chiang, W., Kato, N., Loque, D., Assmann, S.M., Kwak, J.M., Schroeder, J., Rhee, S.Y. and Frommer, W.B. (2010) Frontiers: A Membrane Protein/Signaling Protein Interaction Network for Arabidopsis Version AMPv2. *Front. Physiol.* **1**, 24.

Pradhan-Mitra, P. and Loqué, D. (2014) Histochemical staining of Arabidopsis thaliana secondary cell wall elements. *J. Vis. Exp.* **87**, e51381.

Sparkes, I.A., Runions, J., Kearns, A. and Hawes, C. (2006). Rapid, transient expression of fluorescent fusion proteins in tobacco plants and generation of stably transformed plants. *Nat. Protoc.* **1**, 2019–2025.
